# Macrophages in Calcific Aortic Valve Disease: Paracrine and Juxtacrine Disease Drivers

**DOI:** 10.3390/biom14121547

**Published:** 2024-12-02

**Authors:** Polina Klauzen, Liubov Basovich, Daria Shishkova, Victoria Markova, Anna Malashicheva

**Affiliations:** 1 Laboratory of Regenerative Biomedicine, Institute of Cytology, Russian Academy of Sciences, Saint-Petersburg 194064, Russia.; l.basovich@yandex.com (L.B.); 2 Department of Experimental Medicine, Research Institute for Complex Issues of Cardiovascular Diseases, Kemerovo 650002, Russia; shidk@kemcardio.ru (D.S.); markve@kemcardio.ru (V.M.)

**Keywords:** calcific aortic valve disease, endothelial cells, macrophages, Notch

## Abstract

A significant role in the pathogenesis of CAVD is played by innate immunity cells, such as macrophages. In stenotic valves, macrophages have enhanced inflammatory activity, and the population’s balance is shifted toward pro-inflammatory ones. Pro-inflammatory macrophages release cytokines, chemokines, and microRNA, which can directly affect the resident valvular cells and cause valve calcification. In CAVD patients, macrophages may have more pronounced pro-inflammatory properties, enhanced not only by paracrine signals but also by juxtacrine Notch signaling and epigenetic factors, which influence the maturation of macrophages’ progenitors. In this review, we observe the accumulated data on the involvement of macrophages in CAVD development via paracrine and juxtacrine interactions.

## 1. Introduction

Calcific aortic valve disease (CAVD) is a complex, multifactorial process that leads to the calcification of the valve leaflets, causing hemodynamic obstruction. There is currently no cure for CAVD other than surgical replacement of the affected valve. The disease involves endothelial dysfunction and lipid deposition, inflammation, fibrosis, myofibroblastic/osteoblastic differentiation of valve interstitial cells (VICs), as well as valve calcification, and resembles an atherosclerotic process, but its progress and a final endpoint are distinct [1]. During the development of the disease, valvular endothelial cells (VECs) and VICs interact with each other and with immune cells via paracrine signaling, such as pro-inflammatory cytokines and microRNAs. Juxtacrine Notch signaling has also been shown to play a crucial role in the pathogenesis of CAVD [2]. However, it remains uncertain whether the direct contact between immune cells and valvular cells through Notch receptors and ligands plays a role in CAVD development. In this review, we mainly consider the contribution of macrophages in the pathogenesis of CAVD with special attention to Notch signaling.

## 2. Notch in Driving Calcific Aortic Valve Disease

**Figure 1:**
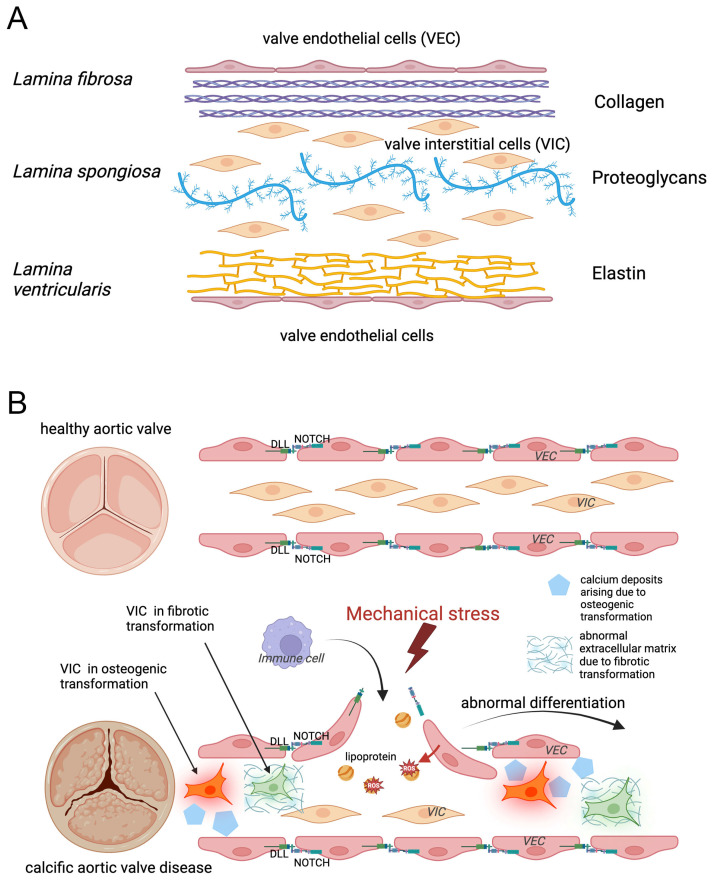
Schematic representation of the pathogenesis of aortic valve calcification in cases where osteoblastic differentiation is involved. (**A**) Simplified structure of a healthy valve. The aortic valve leaflets contain valvular endothelial cells (VECs), valvular interstitial cells (VICs), and the valvular extracellular matrix, where (i) collagen fibrils/fibers (represented by three collagen triple helices in purple) are prominent in the lamina fibrosa, (ii) proteoglycans (in blue) are abundant in the lamina spongiosa, and (iii) elastin fibers (yellow network) are present in the lamina ventricularis. VECs cover the leaflets and regulate valve permeability and homeostasis. VICs are distributed throughout the entire interstitium regulating valve remodeling via synthesis and degradation of valvular extracellular matrix components. Few resident macrophages and dendritic cells as well as a small number of myofibroblast-like VICs, although also present [13] are not depicted here. (**B**) Further simplified representation of cellular events leading to calcific aortic valve disease (CAVD), where the three-valve laminae are not displayed. Notch signaling is essential for maintaining endothelial barrier integrity and proper function (upper figure). Mechanical stress compromises the integrity of the endothelial layer contributing to CAVD development (lower figure): disruption of the barrier function leads to lipoproteins entering the interstitium of the valve leaflets and their oxidation by reactive oxygen species. This process is associated with (i) fibrogenic and/or osteogenic response of VICs, (ii) abnormal differentiation of VECs, and (iii) immune cell infiltration into the leaflet, triggering inflammation and further enhancing the alteration of extracellular matrix composition, ultimately leading to valve dysfunction.

At the beginning of the disease, with mechanical stress, the integrity of VECs is reduced, entailing lipoprotein and immune cell infiltration into the interstitium of valve leaflets [3] (Figure 1). To maintain endothelial barrier function, Notch signaling is essential [2,4]. Accordingly, it has been demonstrated that in the case of inflammation, glycosyl transferase-mediated Notch glycosylation affects cell–cell contacts through altered Notch ligand binding [5]. Therefore, Notch imbalance leads to weakened endothelial function, including reduced cell integrity and abnormal differentiation [6]. Infiltrated lipoproteins are then oxidized by reactive oxygen species produced by dysfunctional endothelial nitric oxide synthase, further augmenting the fibro-osteogenic response of VICs by modulating the nuclear factor kappa B (NF-kB) pathway and activating Notch1 [7]. Oxidized lipids directly stimulate VECs, leading to the upregulation of adhesion molecules such as ICAM-1 and VCAM-1. This allows T-cells and macrophages to infiltrate the tissue, triggering inflammation [8,9,10]. Interestingly, lipid-lowering therapy has no effect on CAVD progression, unlike in atherosclerosis [11,12].

**Figure 2:**
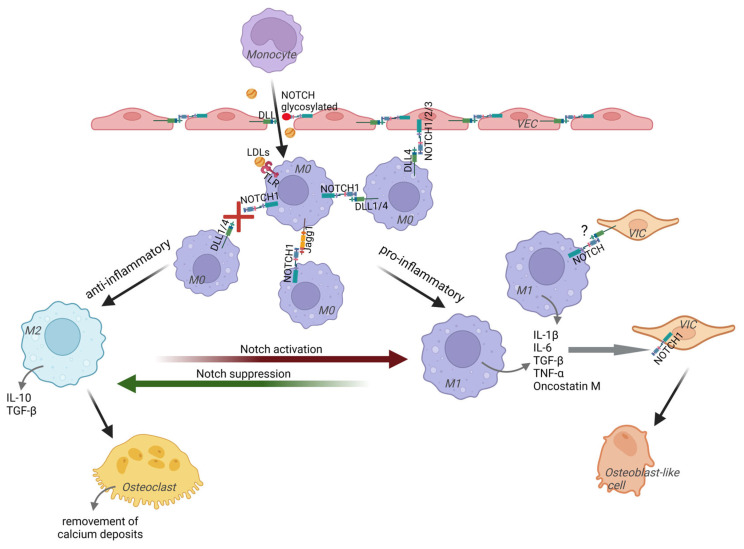
Juxtacrine Notch signaling may play a role at different stages of macrophage involvement in CAVD development. In CAVD, monocytes infiltrate valve tissues due to reduced integrity of VECs and then turn into naive macrophages M0. The reduction in VECs integrity occurs due to mechanical stress and loss of cell–cell Notch contacts after Notch receptor glycosylation observed in inflammatory conditions. In addition to monocytes and other immune cells, low-density lipoproteins (LDLs) also infiltrate the valve and act as osteogenic provocateurs in various ways. LDLs can activate TLRs in macrophages, which in turn induce the expression of Notch receptors and ligands, including Jagged1, Dll1, and Dll4. Activation of the Notch pathway in macrophages can lead to their differentiation into pro-inflammatory M1 phenotype and impact CAVD progression. Furthermore, VECs can induce macrophage M1 transition by losing the Notch4 receptor (while Notch1-3 remain expressed) and increasing the production of Dll4 by both VECs and immune cells. M1 macrophages express cytokines that promote inflammation and influence the osteogenic differentiation of resident valve cells. These cytokines can regulate the Notch signaling pathway by increasing the expression of *NOTCH1*, potentially in VICs as well. Dysregulation of Notch signaling, in turn, stimulates VICs to differentiate into myofibroblast-like cells or osteoblast-like cells. However, it is still unclear whether direct contact between macrophages and VICs via Notch receptors and ligands occurs in CAVD.

Due to the changed integrity of VECs, immune cells also infiltrate into the valve interstitium and become activated. T-cells and monocytes that have penetrated the tissues, which then turn into macrophages, secrete pro-inflammatory cytokines such as tumor necrosis factor (TNF-α), interleukin-1β (IL-1β), and IL-6. These cytokines can modulate the Notch signaling pathway, particularly by increasing the expression of *NOTCH1* [14,15,16] probably also in VICs (Figure 2). It has been demonstrated that cells from patients with CAVD display an imbalanced *NOTCH* profile. Accordingly, it was shown that aortic endothelial cells from CAVD patients have a down-regulated Notch signaling state and fail to activate Notch-dependent endothelial-to-mesenchymal transition (EMT) when stimulated by different Notch ligands or transforming growth factor-β (TGF-β) [17]. Additionally, the Notch-dependent mechanisms in VICs derived from CAVD patients also display altered activity [18]. Numerous studies have shown that dysregulation of the Notch pathway promotes calcification of the aortic valve. However, activation or inhibition of different Notch pathway components can lead to opposite effects on calcification [19,20,21,22,23,24,25,26,27].

Furthermore, with the participation of Notch mechanisms, pro-inflammatory cytokines released by immune cells stimulate VICs to differentiate into myofibroblast-like cells or osteoblast-like cells [28,29]. Myofibroblasts produce extracellular collagen and tenascin-C, which alter the components of the extracellular matrix and promote fibrosis [30]. Osteogenically-differentiated VICs produce bone morphogenetic protein 2 (BMP-2) and osteopontin (OPN), and express Runx2 and osterix (OSX) transcription factors [31,32]. BMP-2 is believed to be a potent initiator of osteoblastic differentiation, while Runx2 acts as the master transcriptional regulator for osteogenesis. Studies have shown that the expression of BMP2 is stimulated by pro-inflammatory cytokine IL-6, and silencing IL-6 results in a reduction in calcium-salt deposition on VICs [33].

Osteogenic differentiation of VICs leads to further calcification and the formation of bone-like structures, although they were found in only 13 to 15% of histologically examined CAVD-affected aortic valves [34,35]. The majority of mineral deposits are acquired as a result of the diffuse precipitation of calcium on cellular debris following apoptosis of VICs. This process is suggested to be regulated by TGF-β [36,37,38]. Interestingly, a pro-calcific cell death other than apoptosis was also identified [39], in which a dramatic release of acidic lipid material derived from degenerating VIC membranes acts as a dominant nucleator of hydroxyapatite crystals, with its final by-products resulting in multitudes of calcospherules, i.e., structures that are commonly detectable in any soft tissue affected by ectopic calcification [40]. Such a degeneration was enhanced when cultures of VICs stimulated with bacterial lipopolysaccharide (LPS) were supplemented with media derived from parallel cultures of LPS-stimulated macrophages. Microcalcification is also promoted by the release of microRNAs, pro-inflammatory, and pro-calcific cargo from extracellular vesicles released by macrophages and VICs [41,42,43,44]. Ecto-nucleotidase ENPP1, which hydrolyzes extracellular adenosine triphosphate and generates inorganic pyrophosphate, contributes to the enhancement of valve calcification [45].

Thus, disruption of Notch-dependent endothelial signaling could contribute to the activation of inflammation-dependent mechanisms of CAVD.

## 3. Macrophages Play a Role in CAVD Progression in Different Ways, Depending on Their Type

It has been shown that both innate immunity and adaptive immunity mechanisms play a major part in the development of CAVD, as well as atherosclerosis. Within the heart valve, around 10–15% of cells evolve from hematopoietic origin [46]. This number increases due to inflammation, as innate immune cells, T-lymphocytes, and B-lymphocytes infiltrate the valve and promote further inflammation, influencing the properties of resident cells. A very special role in disease progression is dedicated to infiltrating monocytes and the tissue macrophages resulting from them. Two gene hubs have been identified in these cells as being closely correlated with CAVD, playing an important role in disease development through immune-related signaling pathways [47].

Macrophages are derived from monocytes after they infiltrate heart valve tissue. Intensive monocyte infiltration has been observed in both early and advanced stages of CAVD [35].

Naïve macrophages (M0) have been shown to be increased in calcified aortic valve tissue samples [48]. These macrophages can acquire a classical (M1, pro-inflammatory) or alternative (M2, anti-inflammatory) phenotype, depending on the activation signals they receive. M2 macrophages produce anti-inflammatory cytokines, such as TGF-β, IL-10, and others, which contribute to the proliferation of regulatory T-cells and immunomodulatory functions [49]. Within the valve, they have the potential to differentiate into osteoclasts, which help remove calcium deposits within the vasculature [50]. In contrast, pro-inflammatory M1 macrophages express enzymes such as nitric oxide synthase and cytokines such as TNF-α, IL-6, and IL-12, which promote inflammation, influence VIC and VEC properties and osteogenic differentiation potential, attract monocytes to the valve, and further propagate the inflammation [51]. IL-6 has been identified as a susceptibility gene underlying CAVD [52]. M1 macrophages accumulate in aortic valvular lesions, which suggests that inflammation induced by these cells may play a pivotal role in cardiovascular calcification [44,53].

The balance of M1 and M2 macrophages may reflect pathological disorders including cancer, infection, and autoimmunity [54,55,56]. Excessive M1 macrophage polarization can lead to atherogenesis, cardiometabolic syndrome, insulin resistance, and adipose inflammation [57,58]. This is due to M1 macrophages’ ability to induce systemic inflammation through the production of IL-6, monocyte chemoattractant protein-1 (MCP-1), and TNF-α, which altogether weaken insulin sensitivity and enhance mechanisms favoring atherosclerotic plaque formation [59,60]. In aortic valve disease, excessive production of inflammatory cytokines has been shown to contribute to a significant shift in the M1/M2 ratio with M2 macrophages being barely observed [48,61]. Therefore, the available data suggest that macrophages play a role in the process of aortic valve calcification.

## 4. Macrophages Play a Signaling Role in Resident Valvular Cells Transformations or Directly Affect the Calcification Process

Macrophages are able to promote microcalcification of the aortic valve by directly releasing microvesicles containing pro-inflammatory and pro-calcific substances [62]. These extracellular vesicles secreted by activated macrophages come into contact with the extracellular matrix, nucleate hydroxyapatite crystals and serve as nucleation sites for microcalcification at the early stages of mineralization [36,63].

In addition, macrophages play a crucial role in the transformation of valvular cells into osteoblast-like cells. This is due to their increased pro-inflammatory activity, which is gained through paracrine and juxtacrine signals that occur during their maturation. Paracrine signaling pathways between macrophages and valve cells have been studied, and they involve pro-inflammatory molecules that drive the transformation of VICs into osteogenic cells, leading to valve calcification. However, juxtacrine interactions between macrophages and residents have not been fully investigated, especially since the role of Notch signaling remains unclear.

VICs are the main osteogenic players in CAVD [64]. VECs have been shown to influence the calcification potential of VICs. In turn, macrophages can influence VEC and VIC properties and induce calcification in the valves. For example, it has been shown that *RUNX2*-expressing osteoblast-like VICs were located significantly closer to macrophages, and exposure to macrophages was associated with the osteogenic calcification of VICs [65]. On the other hand, recently it has been demonstrated that the cellular communication network protein 3 (CCN3), released by macrophages, decreases BMP-2 production and VIC calcification [66].

The great influence of M1 macrophages on valvular cells has been documented, and the conditioned medium from these macrophages enhances VIC calcification [67]. In CAVD, M1 macrophages infiltrate the interstitium of the valve leaflets and release pro-inflammatory cytokines such as IL-1β, IL-6, TGF-β, and TNF-α [68,69]. These cytokines can lead to inflammation of the cardiac valve and influence the process of calcification. It has been shown that TNF-α, IL-1β, and IL-6 promote VIC activation, inhibit the myofibroblast response in VICs, and induce the expression of alkaline phosphatase, eventually leading to the osteogenic differentiation of VICs and valvular calcification [70,71,72,73]. Those cytokines released by pro-inflammatory M1 macrophages can drive a myofibroblast-to-osteogenic switch of VIC phenotypes, which may mediate the transition from fibrosis to calcification during aortic valve stenosis progression [73]. Additionally, TNF-α has been shown to exacerbate valvular inflammation by making VICs more sensitive to toll-like receptor (TLR) activators [74], while TGF-β1 was shown to promote VIC calcification through apoptosis [38]. M1 macrophages produce oncostatin M, which in combination with IL-21 promotes a JAK3/STAT3-dependent osteogenic mechanism in smooth muscle cells [75], which has been confirmed in cultured VICs as well [76]. Furthermore, M1 macrophages express significant amounts of cathepsin S—a cysteine protease that affects the properties of the extracellular matrix [77] and intensifies calcification in CAVD [78]. Activated macrophages also release matrix metalloproteinase (MMP)-1, -2, -3, -9, and -10, which are able to modulate the elasticity of the extracellular matrix (ECM), which in turn determines VICs activation [79].

In addition, M1 macrophages have been shown to promote the calcification of VICs mediated by the microRNA-214/TWIST1 pathway [44]. These macrophages release vesicles containing microRNA-214 (miR-214), which are then delivered to VICs, target TWIST1, and enhance VIC calcification. In this way, macrophages can promote VIC calcification by the delivery of miR-214 to them via macrophage-derived microvesicles and subsequent downregulation of *TWIST1* in VICs.

Macrophages also affect VECs in which TNF-α is known to induce inflammation and oxidative stress [80]. Adult VECs retain the developmental ability to undergo EMT and differentiate into VICs. It has been shown that inflammatory cytokines such as TNF-α and IL-6, released by infiltrating pro-inflammatory macrophages, induce EMT in valve endothelium [81,82,83]. Overall, the data suggest that macrophages contribute to calcification by interacting with the cells of the valve via various signaling mechanisms.

## 5. Macrophages in CAVD Have Strong Pro-Inflammatory Properties

### 5.1. Notch Signaling in Macrophages Regulates Their Pro-Inflammatory Activity

Several studies have reported the expression of Notch1–4 receptors and ligands in human-derived primary macrophages, indicating their ability to both induce and respond to Notch signals [84,85,86]. Notch signaling plays a crucial role in mediating juxtacrine communication between macrophages through interactions with other macrophages and stromal cells. Immunohistochemical and ultrastructural analyses have revealed direct membrane contact between adjacent macrophages in human atherosclerotic plaques [87]. Moreover, upregulation of the Dll4 ligand and multiple components of the Notch signaling pathway within macrophages in these plaques has been demonstrated [88]. These findings suggest that macrophages can function as both Notch signal producers and receivers, responding to external microenvironmental cues. In CAVD, there has been an increased number of macrophages infiltrating and maturing, which is associated with the Notch1 receptor [65].

Notch signaling is considered to be a major regulator of the biological function of macrophages [89]. Activating Notch signaling promotes the differentiation of macrophages into a pro-inflammatory M1 phenotype, whereas blocking Notch signaling polarizes macrophages toward an anti-inflammatory M2 phenotype [90,91,92]. Different cytokines, chemokines, and microRNA are involved in the regulation of macrophage polarization through Notch signaling. IL-37, for instance, inhibits the polarization of macrophages toward M1 by suppressing the *NOTCH1* and NF-kB [93]. MicroRNA miR-148a-3p, on the other hand, promotes the M1 polarization and inhibits the M2 polarization of macrophages upon Notch activation.

Notch signaling in macrophages may also be induced by pathogen-associated molecular patterns (PAMPs) and endogenous molecules, as well as damage-associated molecular patterns (DAMPs), which act via TLRs expressed by macrophages. For example, oxidized low-density lipoproteins (LDLs), which accumulate in the aortic valves of CAVD patients, can activate TLRs in macrophages and trigger an inflammatory response [94] (Figure 2). One mechanism by which TLRs modulate Notch signaling is by inducing the expression of Notch receptors and ligands. Activation of macrophages with TLR ligands has been shown to induce the expression of Notch receptor ligands, including Jagged1, Dll1, and Dll4 [95,96]. Additionally, there is evidence that activation of Notch target genes such as *HES1* and *HEY1* in human primary macrophages can be directly induced by TLR stimulation [97]. Therefore, TLR signaling promotes activation of the Notch pathway in macrophages and can influence their differentiation into the M1 phenotype. This changes the balance between M1 and M2 macrophage populations and impacts CAVD.

### 5.2. Accumulated Lipoproteins Influence on Macrophages’ Inflammatory Potential

The pro-inflammatory properties of macrophages are enhanced by lipoproteins that accumulate in the diseased aortic valve. LDLs can penetrate through the endothelium due to mechanical disruption of contacts between VECs at the initial stages of CAVD development. LDL is the main type of lipoprotein that accumulates in the aortic valve during hyperlipidemia, leading to the recruitment of monocyte-derived macrophages expressing pro-inflammatory genes, which contribute to early-stage aortic valve disease [98]. In the valve, the accumulated LDL molecules become oxidized and are recognized by scavenger receptors of macrophages and then captured. This promotes both inflammation through increased production of IL-1β and TNFα, and inhibition of interferon gamma-induced pro-inflammatory cytokines [99,100,101]. Macrophages in stenotic aortic valves have been shown to express the CLEC4E receptor [102], which recognizes cholesterol crystals. These crystals are then incorporated into the macrophages, where they are degraded, leading to subsequent vascular inflammation and the development of atherosclerosis [103].

Monocyte adhesion with an increase in the inflammatory response may also be enhanced by apolipoprotein ApoC-III, which is known to interact with lipoprotein (a), an intensive CAVD actor synthesized by hepatocytes [104]. ApoC-III is abundantly expressed in the disease-prone fibrosa and accelerates VIC-driven calcification via increased IL-6 production [105].

### 5.3. The Increase in Inflammatory Activity Occurs Even at the Monocyte Stage

Blood monocytes can differentiate into both dendritic cells and macrophages after engagement within inflammatory markers and become highly specialized resident cardiac cells. Monocytes are classified into three subtypes that play significantly different roles in the inflammatory response: classical monocytes (CD14hiCD16null), intermediate monocytes (CD14hiCD16+), and non-classical monocytes (CD14lowCD16hi) [106].

Intermediate monocytes produce the highest amount of reactive oxygen species [107], and when treated with lipopolysaccharides, they produce the largest amounts of TNF-α, IL-1β, and IL-6 [108]. Therefore, they may have greater inflammatory properties than the other two subtypes of monocytes. However, the most abundant monocyte subtypes are classical and non-classical. In conditions of chronic inflammation, such as myocardial infarction and heart failure, the population of intermediate monocytes expands from being almost undetectable to approximately 8% of circulating blood monocytes [109,110]. The same situation of expanding intermediate monocytes is presented in severe aortic valve stenosis valve calcification [46,111]. Exhibiting large amounts of reactive oxygen species and inflammatory markers, interferon-gamma (IFNγ) intermediate monocytes represent the greatest proponent of chronic inflammation within the valve of all monocytes [112].

It is not clear if intermediate monocytes have a discrete biological role or whether they are the unavoidable intermediates in a continuous differentiation from classical into non-classical monocytes. However, they have been found to be increased in certain inflammatory conditions, such as rheumatoid arthritis [113], patients with severe asthma [114], myocardial infarction [109], and loss of kidney function [115,116]. Intermediate monocytes are believed to be indicators of coronary stenosis, as studies have shown that an increase in their number significantly predicts extensive plaque formation [117].

Few studies have characterized monocyte subsets in the setting of CAVD. The study [111] showed that patients with severe CAVD exhibited significantly higher levels of circulating intermediate monocytes, while levels of circulating classical and non-classical monocytes or monocyte activation did not differ compared to controls. The level of circulating intermediate monocytes has been reported to drop after aortic valve replacement [118,119]. Moreover, the amount of intermediate monocytes correlated with worse cardiac function. The relationship between disease severity and monocyte subtypes is still unclear. It remains speculative whether the increased levels of circulating (intermediate) monocytes play a causal role in the pathophysiology of CAVD or are rather a consequence of the disease through hemodynamic changes or valvular inflammation [120]. Although these studies suggest that the phenotype of circulating monocytes is altered in patients with CAVD, a more in-depth exploration of monocyte function and phenotype has not yet been performed.

Inflammatory conditions may cause changes in the signaling pathways of intermediate monocytes. This is shown by Mycobacterium tuberculosis infection, where increased expression of Dll4 was observed in intermediate and non-classical monocytes, leading to activated Notch signaling [121]. Another study found that β-catenin expression is increased in intermediate monocytes in patients with heart failure, which may indicate Wnt/β-catenin signaling intensification [122].

### 5.4. Increase in Pro-Inflammatory Properties at the Stage of Hematopoietic Precursors

Hematopoietic stem cells in the bone marrow give rise to myeloid and lymphoid progenitors; myeloid progenitor cells, in turn, give rise to all leukocytes including monocytes and macrophages [123]. The status of the myeloid progenitor cells and, consequently, the properties of outcoming macrophages are affected by the status of the bone marrow niche. It has been shown that with age and in various diseases, the condition of bone tissue deteriorates, leading to an imbalance in the hematopoietic stem cell niche and an increased production of inflammatory cells in the bone marrow [124]. For example, monocyte/macrophage recruitment from hematopoietic organs may be intensified during the initiation and progression of atherosclerosis [125]. Mice studies have demonstrated that ischemic injury elicits the production of monocytes from the bone marrow, leading to accelerated systemic atherosclerosis [126].

In addition to an increase in number, monocytes/macrophages that are recruited from the bone marrow during cardiovascular disease may have more prominent pro-inflammatory characteristics. This can be influenced by several factors, even during the stage of cell maturation in the bone marrow. Trained immunity and clonal hematopoiesis of indeterminate potential (CHIP) are recently described mechanisms that occur in hematopoietic stem cells. They could potentially contribute to the long-term activation of innate immune cells and influence macrophage activity in CAVD. The discovery of these mechanisms raises the question of whether chronic infiltration of immune cells or the expansion of pathologically altered immune cells within the valve is responsible for the disease progression.

#### 5.4.1. Clonal Hematopoiesis of Indeterminate Potential

Clonal hematopoiesis of indeterminate potential (CHIP) is a phenomenon in which somatic mutations are found in the blood or bone marrow cells without meeting any other criteria for hematologic neoplasia. It becomes more frequent with age and is observed in about 10% of people between the ages of 70 and 80 [127]. In this condition, a big fraction of an individual’s blood cells derives from a single dominant hematopoietic stem cell clone. It was shown that mutations associated with CHIP have effects on macrophages [128]. Since, in a cardiac valve, 10–15% of cells have evolved from hematopoietic origin [46], CHIP may influence the state of macrophages within the aortic valve.

Transcriptomics and immunohistochemistry data confirm that CHIP is common in CAVD patients and its presence is associated with higher mortality [129]. CHIP has also been linked to coronary artery disease and cardio-vascular mineralization; CHIP carriers are four times more likely to experience myocardial infarction than those without it [130,131]. The most common age-related hematopoietic mutations, *TET2* and *DNMT3A*, regulate the inflammatory potential of circulating leukocytes, and their presence is correlated with CAVD and chronic inflammation [131,132]. These mutations are known to increase inflammation by activating the inflammasome complex, leading to increased expression of IL-6 and IL-1β [133]. That is why macrophages in the aortic valves of patients with CAVD may have stronger pro-inflammatory properties and play a role in driving the progression of the disease.

#### 5.4.2. Trained Immunity and CAVD

The concept of trained immunity is also relevant to the question of whether macrophages of CAVD patients are pro-inflammatory and more active. Trained immunity is a phenomenon in which innate immune cells change their properties after contact with DAMPs or PAMPs. This leads to a long-term hyperresponsive state, characterized by an increase in cytokine production [134]. For example, the exposure of human monocytes to oxidized LDLs, uric acid, and adrenaline/noradrenaline induces a trained macrophage phenotype [101,135,136]. Training of tissue macrophages and blood monocytes occurs at the level of myeloid precursors in the bone marrow and that is why hyperresponsive macrophages and monocytes can be found in the organism for a long time. Accordingly, it has been shown that peripheral blood mononuclear cells and bone marrow mononuclear cells from patients with atherosclerosis exhibit a higher capacity for cytokine production following ex vivo stimulation.

The composition of bone marrow was skewed toward myelopoiesis and transcriptome analysis of the hematopoietic cells showed enriched monocyte and neutrophil-related pathways [137,138].

Trained and activated innate immune cells may contribute to pathophysiology and tissue damage. It is known that the development of atherosclerosis, coronary artery disease, and hypercholesterolemia are influenced by trained immunity. Similarly, this mechanism may also be involved in the development of CAVD [139,140,141].

## 6. Conclusions

Cells of innate immunity, such as macrophages, are actively involved in CAVD development. These immune cells interact with resident valvular cells—VICs and VECs—and may contribute to the progression of CAVD. Macrophages communicate with these cells through paracrine and juxtacrine interactions, causing calcification of the aortic valve. The progression of CAVD depends on the pro-inflammatory potential of macrophages. Notch signaling is known to regulate the biological function of macrophages and the osteogenic transformation of VICs, but it is unclear whether direct juxtacrine communication between immune cells and valve cells via Notch receptors and ligands contributes to the progression of CAVD. Therefore, further research into the role of Notch in the interaction between macrophages and valve tissue cells is essential for understanding the pathogenesis of CAVD and for developing therapeutic approaches to targeting inflammation in calcification.
